# An Entropy Based Bayesian Network Framework for System Health Monitoring

**DOI:** 10.3390/e20060416

**Published:** 2018-05-30

**Authors:** Tarannom Parhizkar, Samaneh Balali, Ali Mosleh

**Affiliations:** B. John Garrick Institute for the Risk Sciences, University of California, Los Angeles, CA 90095, USA

**Keywords:** system health monitoring, optimal sensor selection, Bayesian network, information entropy, sensor reliability, multi objective optimization, particle swarm optimization algorithm

## Abstract

Oil pipeline network system health monitoring is important primarily due to the high cost of failure consequences. Optimal sensor selection helps provide more effective system health information from the perspective of economic and technical constraints. Optimization models confront different issues. For instance, many oil pipeline system performance models are inherently nonlinear, requiring nonlinear modelling. Optimization also confronts modeling uncertainties. Oil pipeline systems are among the most complicated and uncertain dynamic systems, as they include human elements, complex failure mechanisms, control systems, and most importantly component interactions. In this paper, an entropy-based Bayesian network optimization methodology for sensor selection and placement under uncertainty is developed. Entropy is a commonly used measure of information often been used to characterize uncertainty, particularly to quantify the effectiveness of measured signals of sensors in system health monitoring contexts. The entropy based Bayesian network optimization outlined herein also incorporates the effect that sensor reliability has on system information entropy content, which can also be related to the sensor cost. This approach is developed further by incorporating system information entropy and sensor costs in order to evaluate the performance of sensor combinations. The paper illustrates the approach using a simple oil pipeline network example. The so-called particle swarm optimization algorithm is used to solve the multi-objective optimization model, establishing the Pareto frontier.

## 1. Introduction

Oil and natural gas can be transported via pipeline at both lower cost and higher capacity when compared to rail and road transit. Pipeline ‘health’ involves unique challenges that include corrosion, leakage, and rupture, impacting transportation efficiency and safety. Through analysis of data gathered from health monitoring sensors and human inspections, pipeline health along with the efficiency and safety of oil and gas transportation can be monitored. However, practicality and cost limits sensing and monitoring, which in turn restricts data availability for health monitoring. This presents itself as a multi-objective sensor selection optimization problem involving the number, location, and type of sensors for a given pipeline network [1]. This paper outlines a sensor selection optimization methodology that leverages the concept of information entropy within a Bayesian framework for system modeling and health monitoring. The overarching aim of this methodology is to obtain more system health information based on an efficient use of information sources.

The problem of optimizing sensor placement has received considerable attention in recent years [2,3]. The approaches of extant optimization models differ in their objective functions, assumptions regarding equation linearity, solution methods, and how they deal with uncertainty. Table 1 lists and categorizes current literature in this regard.

Most of the studies have a single objective function and do not consider system uncertainties. The highlighted cells in Table 1 identify optimization methodology characteristics that are common with the methodology developed herein.

Generally, all sensor optimization methodologies need to deal with trade-offs between sensor reliability, cost, weight, and number. This naturally lends itself to a multi-objective optimization problem involving objective functions with multiple indices. Unlike single objective optimization, multi-objective optimization problems could have multiple optimal solutions and the decision maker can select one of the feasible solutions depending upon the importance of the indices and system limitations [20]. In this paper, an approach that develops Pareto frontier is presented to derive optimal feasible solutions depending on the decision maker’s preference on sensor cost or system information certainty.

A feature of the approach developed herein is the ability to model uncertainties in system model and measurement process. These uncertainties are typically associated with leak location and environmental factors, process conditions, measurement accuracy, etc. The proposed methodology uses Bayesian networks (BNs), integrating representation of the system configuration and information sources, and associated uncertainties. There have been several studies focused on optimizing sensor placement using BN. Flynn et al. [21] define two error types associated with damage detection and use BN to quantify two performance measures of a given sensor configuration. The so called genetic algorithm is used to derive the performance-maximizing configuration. In Li et al. [22] a probabilistic model based on BN is developed that considers load uncertainty and measurement error. The optimal sensor placement is derived by optimizing three distinct utility functions that describe quadratic loss, Shannon information, and Kullback–Leibler divergence. Other studies that focus on BNs include objective functions that describe the minimum probability of errors [23], and the smallest and largest local average log-likelihood ratio [24]. In our study, sensor selection optimization methodology maximizes information metric on system health considering sensor costs. Information entropy quantifies the uncertainty of random variables [25]. 

Minimizing the information entropy decreases the uncertainty. The effectiveness of any model-based condition monitoring scheme is a function of the magnitude of uncertainty in both the measurements and models [26,27]. In [27], it is demonstrated that uncertainty affects all aspects of system monitoring, modelling, and control, and naturally the identification of the optimal sensor combination. The uncertainty stemming from modelling and sensors should therefore be considered in the optimization procedure. 

Uncertainty can be quantified using different metrics. One of the popular metrics of uncertainty quantification is information entropy index. Minimizing the differential entropy decreases the uncertainty or disorder, and hence increases information value [25]. 

A key feature of the method proposed in this paper is that here the optimization is based on answering the following question: which combination of sensor types and locations provides highest amount of information about the reliability metric of interest (e.g., probability of system failure)? The increase or decrease in information is measured by entropy. As a result, different types of sensors can be compared based on their information value, and the optimal sensor combination identified based on a common metric. 

For instance, in a gas pipeline health monitoring context, detectors for temperature, sulfur content, seismic load, human intrusion, corrosion rate, and pipe leakage are compared based on how they change the information on pipe rupture probability (system state), and then the best combination in terms of information gain, considering budget limits, is identified. 

We also note that other research presented in Table 1, focus on the placement of one type of sensor, whereas in the present paper considers simultaneous use of different types sensor as part of optimization on information. For example, in [10], contaminant detectors are optimally placed in a water network in order to reduce the detection time and increase the protected population from consuming contaminated water. 

In Section 2 of this paper, information entropy as it relates to BNs is explained. The proposed sensor selection model based on BNs is presented in Section 3, and the optimization methodology is described in Section 4. In order to illustrate the proposed methodology, it is applied to a very simple oil pipeline example with key features adequate to demonstrate the method and its results. The network model and results are discussed in Section 5. Finally, the study is concluded with an overall discussion on key advantages of the proposed methodology in Section 6.

## 2. An Overview of Information Entropy and BNs

Incomplete information and probabilistic representation of information are generally prevalent in system health monitoring applications. Quantification of the uncertainty is one of the primary challenges for measuring the extent to which you have information regarding a system.

In recent years, several authors have investigated uncertainty representation using the concept of entropy and information theory. Information entropy is used to quantify the average uncertainty of an information source. Let *X* be a random variable with probability distribution of *P*, where *p_i_* is the probability of outcomes $x_{i}\in X$. The information content associated with a particular value of this random variable, as defined by the probability distribution can be calculated as Equation (1) [28].

(1) $$I\left(x_{i}\right)=-\log p_{i}$$

The information function, computes the amount of information (measured in bits) conveyed by a particular state. The expected value of information is the information entropy function H(X), which is calculated by Equation (2).

(2) H(X)=E[I(X)]

The expression for information entropy is developed further in Equation (3) for discrete random variables [28].

(3) $$H\left(X\right)=\overset{n}{\underset{i=1}{\sum }}p_{i}\times I\left(x_{i}\right)=-\overset{n}{\underset{i=1}{\sum }}p_{i}\times \log p_{i}$$

Generalizing to continuous random variables, the information entropy of the random variable X is defined as

(4) $$H(X)=-\int_{}^{}\left(f(x)\times \log f(x)\right)dx$$

**Figure 1:**
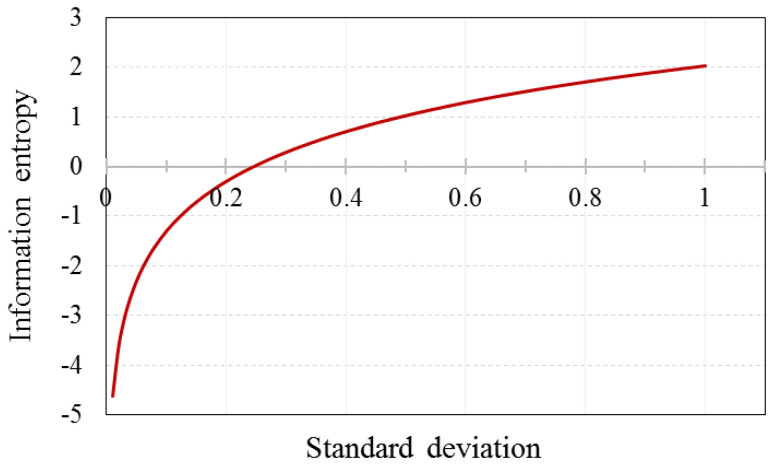
Information entropy as a function of dispersion for a normally distributed variable.

The information entropy increases with respect to the data uncertainty of a random variable. It also increases as the ‘dispersion’ of a random variable increase, as illustrated in Figure 1 for the standard deviation of a normal distribution function $f(x|\mu ,\sigma^{2})$.

In methodology proposed below, the distribution of BN (or system) state variables [10] can be constructed by domain experts or synthesized automatically from system operating data. Probability distributions of these state variables convey information uncertainty of the system status. In a BN with single valued probabilities of random variables $x_{i}$ and joint probability, P, given by

(5) $$P(x_{1},x_{2},\ldots ,x_{n})=\overset{n}{\underset{i=1}{\Pi }}p(x_{i}|y)$$

the information entropy is calculated from Equation (6), [25,29].

(6) $$H(x_{1},x_{2},\ldots ,x_{n})=-\sum_{i=1}^{n}p(x_{i})\times \log p(x_{i})$$

## 3. Sensor Selection Optimization Based on Information Entropy in BNs

**Figure 2:**
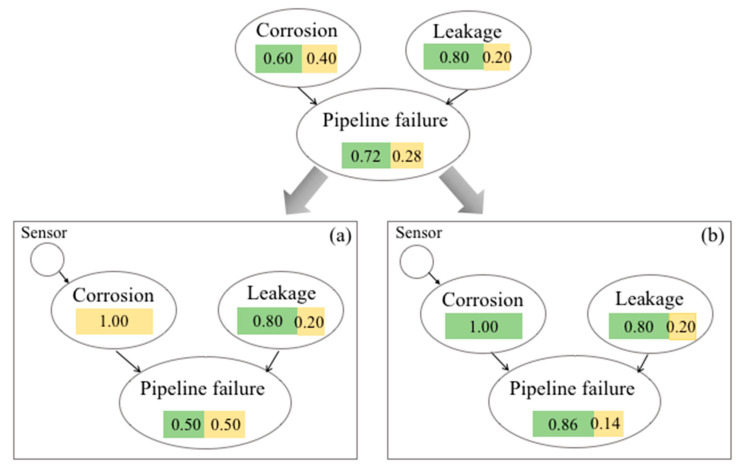
BN evidence scenarios with corrosion sensor. (**a**) no corrosion; (**b**) corrosion.

The assessment of system health and condition is based on our understanding of the BN node state variables, which are represented by their joint probability distributions. Figure 2 illustrates the inference engine of a simple three-node BN in which each node has two states: success (green) and failure (yellow). The joint probabilities of this network are presented in Table 2.

According to Table 2, the failure probability of the pipeline node can be calculated as

(7) $$\begin{array}{cc} P(f) & =P(f|nc.nl)\times P(nc)\times P(nl)+P(f|nc.l)\times P(nc)\times P(l)\\ & \;+P(f|c.nl)\times P(c)\times P(nl)+P(f|c.l)\times P(c)\times P(l)\\ & =0.10\times 0.60\times 0.80+0.30\times 0.60\times 0.20+0.40\times 0.40\times 0.80+0.90\times 0.40\times 0.20=0.28 \end{array}$$

The probability of the safe state of pipeline node is then equal to P(s)=1−P(f)=0.72, which is presented in Figure 2 with a green box.

As a sensor is placed on a node, the posterior probability distribution of BN state variables can be computed using evidence nodes. Evidence contains information regarding a set of random variables, and the posterior probability of monitored state $x_{e}$ of an evidence node is equal to one at the observed value. 

(8) $$p(x_{e}^{*})=1$$

For instance, in Figure 2, sensor on corrosion node updates the probabilities of this node. The sensor can detect two states of ‘corrosion’ (Scenario (a)) and ‘no corrosion’ (Scenario (b)) in the pipeline. For instance, in Scenario (a), the probability of corrosion is updated to 1, and the probability of no corrosion state is updated to 0, as presented in Figure 2a. Consequently, the posterior probability distribution of BN states is updated. 

In this paper, placing a sensor at a particular place in a system makes the corresponding node an evidence node. Moreover, it is assumed that the sensor reports all states of the evidence node. Therefore, total system information entropy can be calculated based on the probability distribution of the state of the evidence node as shown in Equation (9)

(9) $$H(x)=-\sum_{k=1}^{k^{*}}\left(p_{k}\times \sum_{j=1}^{m}\sum_{i=1}^{n_{j}}p(x_{ijk})\times \log p(x_{ijk})\right)$$

Here, $n_{j}$ is the number of node states, m is the number of BN nodes and $k^{*}$ is the number of possible evidence observations scenarios based on selected sensor and $p_{k}$ is its probability. 

Based on the prior knowledge of the state variables’ probability distribution, the total system information entropy is 0.77, which is equal to the sum of the all individual node information entropies. 

(10) $$\begin{array}{l} H(x)=-\sum_{j=1}^{3}\sum_{i=1}^{2}p(x_{ija}).\log p(x_{ija})=0.60\times \log0.60+\\ 0.40\times \log0.40+0.80\times \log0.80+0.20\times \log0.20+0.72\times \log0.72+0.28\times \log0.28=0.77 \end{array}$$

By placing a sensor on the corrosion node, the system information entropy is calculated using all possible sensor observations (evidence). In the example below, possible sensor evidence observations are two scenarios (a) and (b), and the total system information entropy would be 0.44

(11) $$\begin{array}{cc} H(x) & =-p_{a}\sum_{j=1}^{3}\sum_{i=1}^{2}p(x_{ija})\log p(x_{ija})-p_{b}\sum_{j=1}^{3}\sum_{i=1}^{2}p(x_{ijb})\log p(x_{ijb})\\ & =0.40\times 0.52+0.60\times 0.39=0.44 \end{array}$$

As can be seen, corrosion node has two states. Therefore, two evidence observation scenarios are possible. Scenario (a) is the failure state of the corrosion node (sensor does not detect corrosion) and scenario (b) is its success (sensor detects corrosion). In Equation (11), $p_{a}$ and $p_{b}$ are the prior probabilities of corrosion node states for (a) and (b) scenarios, respectively.

### 3.1. Information Value

In the example of Figure 2, it is assumed that information associated with all nodes have the same weight (value). The weight of a node information reflects the extent to which it informs a subsequent decision, or yields value to an organization or activity, such as the modification of inspection plan. In most cases, the expected cost of failure of different system elements (represented by BN nodes) are not identical. Consequently, the information entropy importance of different nodes would not be equal. To accommodate such situations, information entropy of each node can be weighted via Equation (12).

(12) $$w_{j}=m^{\prime }\frac{{\left(\mathrm{expected}\;\mathrm{cost}\;\mathrm{of}\;\mathrm{failure}\right)}_{j}}{\sum_{j=1}^{m^{\prime }}\mathrm{expected}\;\mathrm{cost}\;\mathrm{of}\;\mathrm{failure}}$$

where $m^{\prime }$ is the number of nodes with different information value. The weights sum to one. The total system information entropy is then calculated using Equation (13).

(13) $$H(x)=-\sum_{k=1}^{k^{*}}\left(p_{k}\times \sum_{j=1}^{m}w_{j}\times \sum_{i=1}^{n}p(x_{ijk})\times \log p(x_{ijk})\right)$$

### 3.2. Sensor Reliability and Measurement Uncertainty

BN state variable probability distributions are continually updated using sensor data, noting that they can be uncertain [25]. This uncertainty may be inherent to the process of gathering data (condition variability and human observation uncertainty) or it may stem from the sensor uncertainty. To consider these uncertainties, sensors can be represent as ‘soft evidence’ nodes, which carry two additional piece of information: the operational mode and the probability of its occurrence [30].

**Figure 3:**
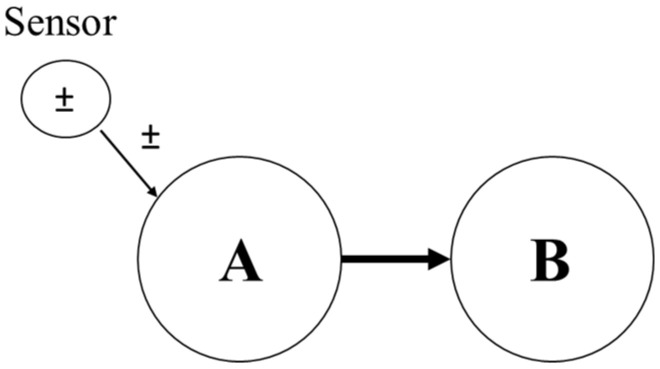
Considering sensor as a soft evidence in BN.

The state of knowledge about BN ‘soft evidence’ nodes are usually modelled by probability distributions using Jeffrey’s rule [29]. The posterior probability of node B’s state variable presented in Figure 3 is defined as

(14) $$P(B)=\sum_{i}P(B|A_{i})\times P(A|S_{i})$$

where, P(A|S) is the conditional probability of A given ‘soft evidence’, and P(B|A) is the conditional probability of B given A, before evidence. 

## 4. Optimization Methodology

### 4.1. Problem Formulation

The sensor selection optimization problem involves the competing goals of maximizing information, minimizing sensors cost, and optimizing physical constraints (such as size and weight limitations). In an m node BN with $T_{j}$ possible sensor types for the *j*-th node under consideration, and $M_{T_{j}}$ models for the sensor type $T_{j}$, the number of possible sensor selection combinations is

(15) $$\prod_{j=1}^{m}\left(T_{j}\times M_{T_{j}}+1\right)$$

The multi-objective optimization approach used herein is based on an objective function that contains two weighted indices representing sensor cost and information entropy as described in Equation (16).

(16) $$Min\;\left[\omega_{1}\times C(y)+\omega_{2}\times H(y)\right]$$

where C(y) is the cost of a particular sensor configuration, *H*(*y*) is system information entropy, and $\omega_{1},\omega_{2}$ are the weighting factors of the cost and system information entropy, respectively. Using weight values is one of the methods for transforming multi-objective to single-objective problems. In this approach, different weight values are considered, and a different solution is derived for each of the used weights. Pareto front is then obtained based on combination of these solutions.

The objective function can be subject to several constraints. For instance, Equation (17) imposes a limitation on budget or total sensor cost, and Equation (18) imposes a minimum acceptable level of system health information.

(17) $$C(y)\leq C^{\max}$$

(18) $$I(y)\geq I^{\min}$$

### 4.2. Solution Approach

A multi objective optimization problem usually has a set of solutions that is known as the Pareto-optimal set. Each Pareto optimal solution in an optimal set which represents a compromise between objective functions, acknowledging that the objective functions cannot be all simultaneously improved. Different solution approaches, including exact and heuristic, have been proposed to solve multi-objective optimization problems once the multi-objective problem is transformed into single objective case. Linear or small size problems can be solved using exact solution algorithms. These algorithms include gradient search method, dynamic programming, and branch-and-bound algorithm which is mainly used in linear mixed-integer problems. Heuristic methods such as artificial bee evolutionary programming (EP), genetic algorithm (GA), and particle swarm optimization (PSO) are generally used for larger or nonlinear problems [31,32]. 

The most commonly used heuristic methods are population-based evolutionary techniques that are inspired by evolution in nature. The subject now includes GA and PSO. These algorithms stem from the very basic description of biological systems. Evolutionary techniques are classified as stochastic search algorithms for global optimization problems, which have found many engineering and industrial applications. The GA and PSO algorithms have been compared in [33]. Results indicate that PSO is more computationally efficient as it uses fewer number of function evaluations.

PSO is inspired by simulations of social behaviors. PSO shares many similarities with evolutionary computation techniques such as GAs, including initialization with a population of random solutions and search for optima by updating generations. However, unlike GA, PSO has no evolution operators such as crossover and mutation. In PSO, potential solutions, called particles, fly through a problem space by following the current optimum particles. In general, compared with GAs, the advantages of PSO is that it is easy to implement and there are few parameters to adjust. Recent studies of PSO indicate that although the standard PSO outperforms other EAs in early iterations, it does not improve the quality of solutions as the number of generations is increased. This means that it can converge with fewer iterations, and as a result the solution time is shorter that other EAs [34,35].

**Figure 4:**
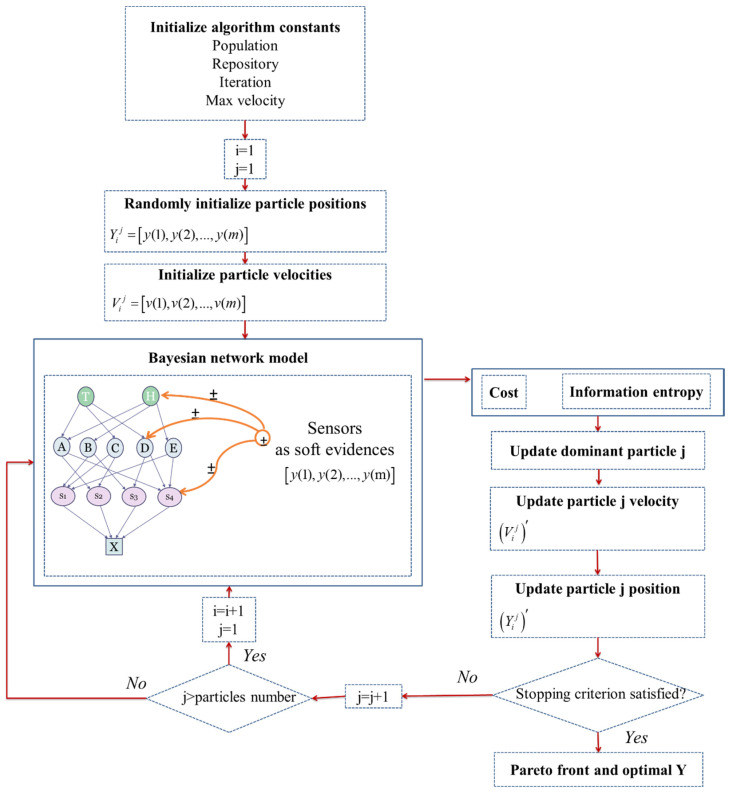
Data flow of sensor selection optimization procedure based on IMOPSO.

The proposed optimization model in this study is an integer nonlinear problem. The PSO method is a better solution approach for this problem in comparison with other algorithms due to its simplicity of calculations, not needing mutation and overlapping calculations, and high speed of convergence in nonlinear optimization problems [35,36]. Figure 4 depicts the data flow diagram of the developed integer multi objective particle swarm optimization (IMOPSO) algorithm that can be used in sensor selection optimization problems based on BNs. The IMOPSO algorithm randomly initializes or creates a population of state variable sets. Each state variable set is called a ‘particle’ Y=[y(1),y(2),…,y(m)] in which y(j) is the *j*-th node’s state variable (sensor status). Including sensor existence, sensor types $T_{j}$, and models $M_{T_{j}}$. The particle moves through the solution space following some basic formulae in search of a global optimum. The velocity of each particle V=[v(1),v(2),…,v(m)] at each generation of movement changes based on the last optimum particles [37]. After several generations, only the ‘most optimal’ particles can transmit information to other particles, making the optimization very fast in comparison to other evolutionary techniques [38,39].

## 5. Application Example and Numerical Evaluation

In the following, the proposed methodology is illustrated with a very simple example that has the key characteristics to show how the technique works and what types of results could be expected. 

### 5.1. Problem Statement

**Figure 5:**
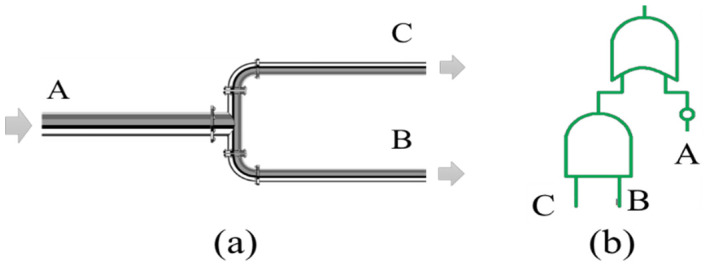
(**a**) Studied oil pipeline schematic; (**b**) fault tree of the pipeline.

The problem to be addressed is sensor selection in an oil pipeline network. The pipeline is divided into three segments with different characteristics. The general structure of the three-segment pipeline and a corresponding fault tree are illustrated in Figure 5a,b, respectively.

**Figure 6:**
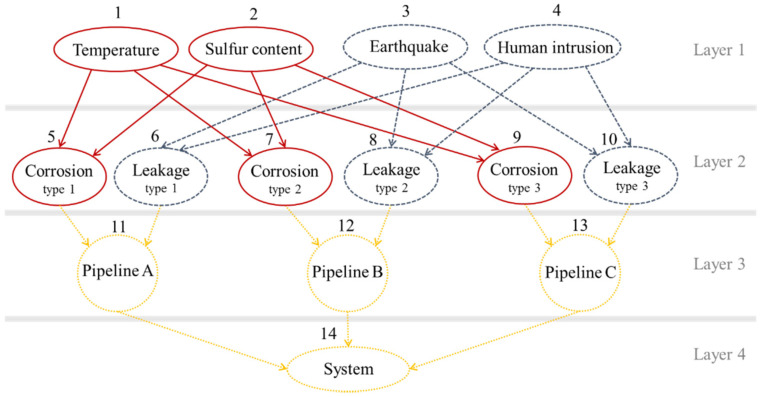
BN of the studied oil pipeline network.

The fault tree gates are mapped into the BN and event likelihoods are defined using a conditional probability table. Figure 6 shows a four-layer BN of the exemplar oil pipeline network. The first layer of nodes are external causes of system degradation, here ambient temperature, chemical content, earthquake shock, and human intrusion. The second layer nodes primarily consist of corrosion and leakage. The failure mechanisms are assumed to be independent. The third layer presents the health status nodes of the three pipeline segments. 

Table 3 and Table 4 are examples of the conditional probability tables for corrosion mechanism. 

The effect of sensor uncertainties is considered in the proposed Bayesian model by assuming that all nodes are soft nodes which are inherently uncertain. Therefore, the reliability of the sensor, which is highly dependent on its cost, is assumed to affect the information certainty. Table 5 presents sensors relative cost and reliability in the studied case. 

### 5.2. Results and Discussion

**Figure 7:**
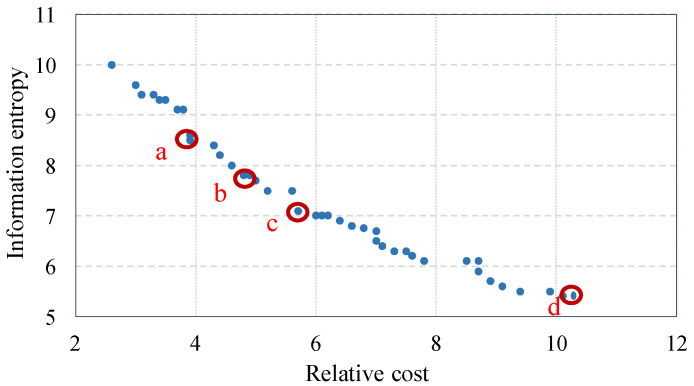
Pareto front of optimal sensor combinations.

The population size, maximum number of iterations, and size of the external repository should be determined for the proposed IMOPSO algorithm. In general, the population size and number of iterations have an inverse relation, as a smaller population size requires higher number of iterations and vice versa. In this study, a population size of 50 is used and the maximum number of iterations is guided by convergence of the results. In addition, the maximum size of repository is set to 100 and a variable-size repository is initially set to 5% of maximum size of repository, and then increased in a stepwise manner until it reaches the maximum size of repository. The results converge well when repository of 50 is used. Figure 7 is the Pareto frontier of locally optimum sensor selection. Each point on the curve has no other sensor combinations where both cost and entropy are better.

As illustrated in Figure 7, relative cost ranges between 2.6 and 10.5 corresponding to information entropy ranging between 5.4 and 10. The range of Pareto optimal solutions along the Pareto frontier provides the decision maker ample flexibility to identify an optimum cost-effective combination, while maintaining acceptable system information entropy. Further, the Pareto frontier illustrates the relationship between marginal information entropy and relative cost at optimum sensor combination. 

Table 6 describes selected optimal sensor combinations on the Pareto frontier illustrated in Figure 7. As can be seen, higher budgets permit higher reliability (Model 3 rather than 2 or 1). 

**Figure 8:**
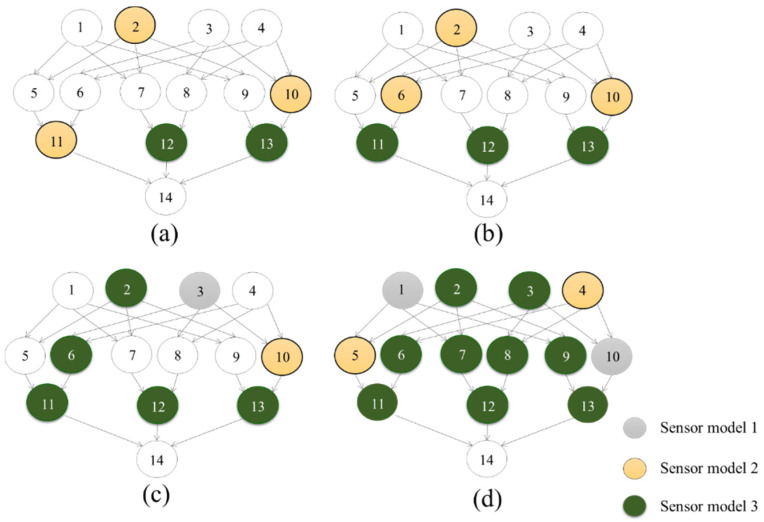
Optimal sensor combinations of selected optimal scenarios. (**a**) Scenario a; (**b**) Scenario b; (**c**) Scenario c; (**d**) Scenario d.

Figure 8 illustrates the selected optimal sensor locations (from Figure 7 and Table 6) with respect to the BN. It can be seen that the optimization methodology preferences sensors at the third layer.

The optimization process up until this point has assumed that the information about each of three pipeline segments have the same value. This may not be the case in practice. For example, one pipe segment may have greater difficulties associated with maintenance crew access, making failure more expensive. To make the optimization more robust, information value can be weighted based on failure cost, which will be a function of both reliability and repair cost. 

Key statistics (in the form of percentiles) of the resultant segment failure frequencies of pipeline segments are presented in Table 7. It can be seen that segment A is the least reliable, followed by segments B and C, respectively.

Taking into account the failure cost of individual pipe segments, the ‘rank’ of segment importance changes as seen in Table 8. 

The coefficient weight of information entropy of each node can be calculated from Equation (10), and system total entropy is evaluated based Equation (19).

(19) $$\begin{array}{l} H(x)=\\ -\sum_{k=1}^{r}\left(p_{k}\times \left(\sum_{j=1}^{10}\sum_{i=1}^{n}H_{ijk}+1.79\times \sum_{i=1}^{n}H_{i11k}+0.56\times \sum_{i=1}^{n}H_{i12k}+0.65\times \sum_{i=1}^{n}H_{i13k}+\sum_{i=1}^{n}H_{i14k}\right)\right) \end{array}$$

**Figure 9:**
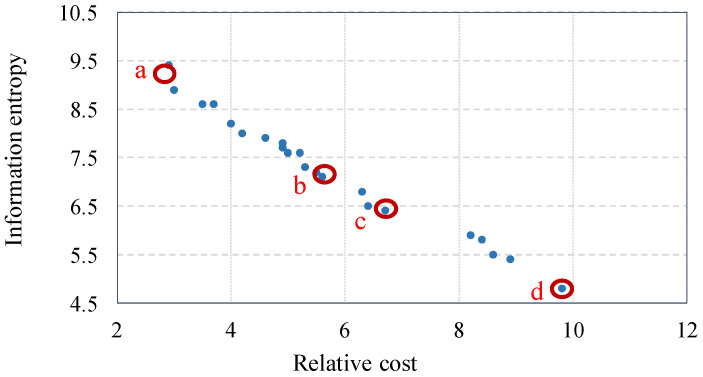
Pareto frontier of locally optimal sensor combinations.

Figure 9 illustrates the Pareto front considering information value of pipeline segments in the optimization procedure. 

The selected optimal combinations on the Pareto front are presented in Table 9.

**Figure 10:**
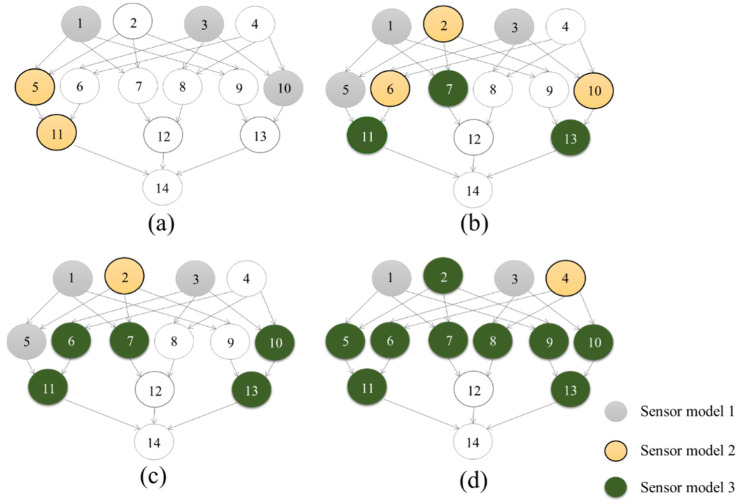
Optimal sensor combinations of selected optimal scenarios considering information value. (**a**) Scenario a; (**b**) Scenario b; (**c**) Scenario c; (**d**) Scenario d.

The selected optimal sensor combinations are illustrated as they relate to the BN in Figure 10. As can be seen, the optimal combinations differ from Figure 8. In this scenario, sensors are placed reflecting information value of each node, and consequently as is shown, the minimum number of sensors tend to be those that provide more information about node 11 (pipeline A), followed by node 13 (pipeline C). This reflects the ‘importance rank’ from Table 8.

## 6. Concluding Remarks

Optimal sensor selection for system health monitoring is a generally well explored problem in industrial systems, but there is scope for it to be developed further. This paper proposes a new methodology for sensor selection optimization based on information gain and sensor cost. The novelty of the methodology lies in the application of the information entropy using BN model of the system and information sources in a way that incorporates sensor and measurement uncertainties. The developed methodology is illustrated using a very simple oil pipeline network, producing several optimal sensor combinations. The PSO algorithm was used to solve the multi-objective optimization problem, producing a Pareto frontier. Results show that the proposed methodology is effective in sensor selection optimization problems with multiple criteria that involve uncertainty. Furthermore, the information value of Bayesian nodes is weighted regarding nodes failure costs, and results indicate that sensor optimal combinations are highly affected by weighting information value.

## Figures and Tables

**Table 1:** Sensor placement optimization literature summary.

Objective Function	Linearity	Uncertainty	Solution Method	System under Study	Ref.
Single	Linear	Not considered	Cutting-plane approach, semi-definite programming approach	Framework	[4]
Single	Linear	Not considered	Mixed integer linear programming solver	Oil Pipeline	[5]
Single	Linear	Not considered	Worst case energy balance strategy	Oil Pipeline	[6]
Single	Linear	Considered	CPLEX	Gas detector	[7]
Single	Nonlinear	Not considered	Genetic algorithm, ant colony algorithm	Oil Pipeline	[8]
Single	Nonlinear	Not considered	Dynamic programming, simulated annealing, Particle swarm optimization algorithm, ant colony optimization algorithm	High-speed rail	[9]
Multi	Nonlinear	Not considered	Greedy algorithm	Water network	[10]
Single	Nonlinear	Considered	Bayesian approach	Framework	[11]
Single	Nonlinear	Considered	Bayesian approach	Framework	[12]
Single	Nonlinear	Considered	Stochastic decomposition algorithm	Water network	[13]
Single	Nonlinear	Considered	Bayesian approach, genetic algorithm	Framework	[14]
Single	Nonlinear	Considered	Gradient search method	Transport-reaction process	[15]
Single	Nonlinear	Considered	Genetic algorithm	Power distribution system	[16]
Multi	Nonlinear	Considered	Annealing algorithm	Shell structure	[17]
Multi	Nonlinear	Considered	Hybrid greedy randomized adaptive search procedure	Vehicular network	[18]
Multi	Nonlinear	Considered	Genetic algorithm	Framework	[19]

**Table 2:** Joint probabilities of the BN presented in Figure 2.

State	Probability
P( failure| no corrosion, no leakage)=P(f|nc,nl)	0.10
P( failure| no corrosion, leakage)=P(f|nc,l)	0.70
P( failure| corrosion, no leakage)=P(f|c,nl)	0.60
P( failure| corrosion, leakage)=P(f|c,l)	0.90

**Table 3:** An example of the conditional probability table for corrosion mechanism.

Temperature (Celsius)	H_2_S (ppm)	Corrosion Pipeline A		Corrosion Pipeline B		Corrosion Pipeline C	
		Yes	No	Yes	No	Yes	No
20–40	0–1000	0.3	0.7	0.1	0.9	0.2	0.8
20–40	1000–10,000	0.4	0.6	0.2	0.8	0.3	0.7
40–60	0–1000	0.35	0.65	0.3	0.7	0.4	0.6
40–60	1000–10,000	0.45	0.55	0.7	0.3	0.8	0.2
60–80	0–1000	0.4	0.6	0.6	0.4	0.7	0.3
60–80	1000–10,000	0.7	0.3	0.8	0.2	0.9	0.1
80–100	0–1000	0.45	0.55	0.3	0.7	0.4	0.6
80–100	1000–10,000	0.8	0.2	0.7	0.3	0.8	0.2

**Table 4:** An example of the conditional probability table for leakage.

Earthquake (Richter)	Human Intrusion (kJ)	Leakage Pipeline A		Leakage Pipeline B		Leakage Pipeline C	
		Yes	No	Yes	No	Yes	No
3–4	0–10	0.3	0.7	0.2	0.8	0.1	0.9
3–4	>10	0.7	0.3	0.6	0.4	0.5	0.5
4–5	0–10	0.75	0.25	0.7	0.3	0.65	0.35
4–5	>10	0.8	0.2	0.7	0.3	0.6	0.4
5–6	0–10	0.85	0.15	0.8	0.2	0.75	0.25
5–6	>10	0.95	0.05	0.9	0.1	0.85	0.15

**Table 5:** Reliability and relative cost of sensors.

Node Number	Sensor Type	Sensor Model	Reliability	Relative Cost
1	Temperature	1	0.85	0.5
		2	0.9	0.7
		3	0.95	0.9
2	Sulphur detector	1	0.85	0.5
		2	0.9	0.7
		3	0.95	0.9
3	Earthquake detector	1	0.85	0.5
		2	0.9	0.7
		3	0.95	0.9
4	Human intrusion detector	1	0.85	0.5
		2	0.9	0.7
		3	0.95	0.9
5	Corrosion detector	1	0.85	0.5
		2	0.9	0.7
		3	0.95	0.9
6	Leakage detector	1	0.85	0.5
		2	0.9	0.7
		3	0.95	0.9
7	Corrosion detector	1	0.85	0.5
		2	0.9	0.7
		3	0.95	0.9
8	Leakage detector	1	0.85	0.5
		2	0.9	0.7
		3	0.95	0.9
9	Corrosion detector	1	0.85	0.5
		2	0.9	0.7
		3	0.95	0.9
10	Leakage detector	1	0.85	0.5
		2	0.9	0.7
		3	0.95	0.9
11	Failure detector of pipeline A	1	0.85	0.5
		2	0.9	0.7
		3	0.95	0.9
12	Failure detector of pipeline B	1	0.85	0.5
		2	0.9	0.7
		3	0.95	0.9
13	Failure detector of pipeline C	1	0.85	0.5
		2	0.9	0.7
		3	0.95	0.9

**Table 6:** Selected optimal combinations on the Pareto front.

No.	Information Entropy	Relative Cost	Sensors Combination (Sensor, Model)	Information Uncertainty
a	8.6	3.9	(2, 2), (10, 2), (11, 2), (12, 3), (13, 3)	0.31
b	7.8	4.8	(2, 2), (6, 2), (10, 2), (11, 3), (12, 3), (13, 3)	0.305
c	7.1	5.7	(2, 3), (3, 1), (6, 3), (10, 2), (11, 3), (12, 3), (13, 3)	0.298
d	5.2	10.5	(1, 1), (2, 3), (3, 3), (4, 2), (5, 2), (6, 3), (7, 3), (8, 3), (9, 3), (10, 1), (11, 3), (12, 3), (13, 3)	0.285

**Table 7:** Failure frequencies of three segments (per km·year) [40].

	5%	50%	95%
Pipeline A	4.6× 10^−4^	2.28 × 10^−3^	10.66 × 10^−3^
Pipeline B	1.82 × 10^−4^	1.75 × 10^−3^	7.95 × 10^−3^
Pipeline C	1.47 × 10^−4^	1.73 × 10^−3^	5.97 × 10^−3^

**Table 8:** Expected cost of failure and information value rank of three segments [40].

Pipeline Segment	Failure Cost ($K)	Expected Annual Cost of Failure ($K/km·year)	Rank
Pipeline A	5100	11.6	1
Pipeline B	2095	3.67	3
Pipeline C	2425	4.2	2

**Table 9:** Selected optimal combinations on the Pareto front.

No.	Information Entropy	Relative Cost	Sensors Combinations (Sensor, Model)	Information Uncertainty
a	9.4	2.9	(1, 1), (3, 1), (5,2), (10, 1), (11, 2)	0.318
b	6.8	6.3	(1, 1), (2, 2), (3,1), (5, 1), (6, 2), (7, 3), (10, 2), (11, 3), (13, 3)	0.295
c	6.4	6.7	(1, 1), (2, 2), (3, 1), (5, 1), (6, 3), (7, 3), (10, 3), (11, 3), (13, 3)	0.291
d	4.8	9.8	(1, 1), (2, 3), (3, 1), (4, 2), (5, 3), (6, 3), (7, 3), (8, 3), (9,3), (10, 3), (11, 3), (13, 3)	0.280
